# Chronic intestinal immune activation reveals separable impacts of inflammation and barrier loss on hallmarks of ageing

**DOI:** 10.1371/journal.pone.0342910

**Published:** 2026-02-13

**Authors:** Jeanette Alcaraz, Charlotte Keyse, Charles Hall, David W. Walker, David P. Doupé, Rebecca I. Clark

**Affiliations:** 1 Department of Biosciences, Durham University, Durham, United Kingdom; 2 Department of Integrative Biology and Physiology, University of California, Los Angeles, California, United States of America; University of Palermo, ITALY

## Abstract

Inflammaging is considered a driver of age-associated pathology across tissues. Similarly, intestinal permeability is a feature of ageing and underlies a range of inflammatory and age-related diseases. Increased intestinal permeability has been described as both a cause and a consequence of inflammation. Both intestinal permeability and inflammation are closely associated with microbial dysbiosis, epithelial dysplasia and mortality but dissecting the complex interplay between these phenotypes remains challenging. Here we genetically induce intestinal immune activation in *Drosophila* and stratify animals by their intestinal barrier status using the Smurf assay. We demonstrate that intestinal barrier failure has a distinct impact on the microbiota. Further, immune activation, both within the intestine and systemically, drives intestinal barrier failure and mortality even in the absence of the microbiota. Importantly, immune-induced intestinal barrier failure takes time to develop and is closely associated with the onset of mortality. Our work adds to building evidence that the impact of intestinal permeability on the microbiota and on animal health needs to be considered independently of its relationship with inflammation.

## Introduction

Chronic low-grade inflammation, or “inflammaging,” has been proposed as a unifying mechanism connecting age-associated pathologies across tissues [1]. Elevated intestinal permeability has also been observed in a range of inflammatory and age-related diseases [2,3] and is increasingly recognized as a physiological feature of ageing. Intestinal permeability has been linked to systemic inflammation, microbial dysbiosis, and mortality across species [4–7]. However, the directionality of the association between intestinal permeability and inflammation, and the role of the intestinal microbiota in this relationship, remains unclear. Specifically, it is not well established whether intestinal permeability initiates inflammation and pathology or arises as a secondary consequence [3,8].

It is well established that inflammation, via cytokines such as TNF-α and IFN-γ, can disrupt epithelial tight junctions and promote barrier leakiness [8,9]. However, several longitudinal and mechanistic studies suggest that increased intestinal permeability may precede and contribute to the development of inflammatory disease. In human cohorts, elevated intestinal permeability has predicted subsequent development of Crohn’s disease [10,11]. Similarly, age-associated increases in intestinal permeability and tight junction remodeling have been documented in non-human primates [12], indicating that epithelial barrier deterioration may occur independently of systemic inflammation. Evidence from multiple species supports that disruptions in junctional integrity and altered barrier function can be early features in both disease progression and ageing processes [13].

This bidirectional relationship between inflammation and intestinal permeability is further complicated by a close association with microbial dysbiosis and epithelial dysplasia. In model organisms these phenotypes are often co-regulated and experimentally inseparable, whereby interventions targeting one phenotype frequently impact the others [6,14–19]. Intestinal permeability, age-related inflammation and increased microbial loads have been documented in aged flies, mice and monkeys [6,7,14]. Germ free flies and mice show reduced age-related inflammation and improved barrier function [6,14] suggesting that the aged microbiota may drive both inflammation and intestinal permeability. However, studies in *Drosophila* have also demonstrated a role for innate immune activation in driving microbial dysbiosis. Immune signalling is normally tightly controlled by multiple negative regulators. A loss of negative regulation of immune signaling results in chronic immune activation, microbial dysbiosis and shortened lifespan [16]. Conversely, restoration of immune suppression can prevent age-related microbial overgrowth and improve lifespan [17,18].

Dissecting these complex interrelationships requires experimental approaches that allow the separation of the effects of immune activation, epithelial dysplasia, intestinal permeability and microbial dysbiosis. Such approaches are essential if we are to move toward a causal understanding of the role of these phenotypes in age-related decline.

In *Drosophila*, the “Smurf” assay offers a well-established non-invasive method to stratify animals based on intestinal permeability. However, to date studies focused on the role of immune activation in driving microbial dysbiosis, epithelial dysplasia and mortality that measure or account for intestinal permeability are lacking. In this study, we use a *Drosophila* model of intestinal innate immune activation to investigate the relationship between immune activation, intestinal barrier dysfunction and associated phenotypes. We stratify animals by their intestinal barrier status allowing us to clarify the relationships between innate immune signaling, intestinal permeability, microbial dysbiosis, epithelial dysplasia and mortality.

## Results

### Intestinal overexpression of PGRP-Lc drives intestinal barrier loss and mortality

**Fig 1:**
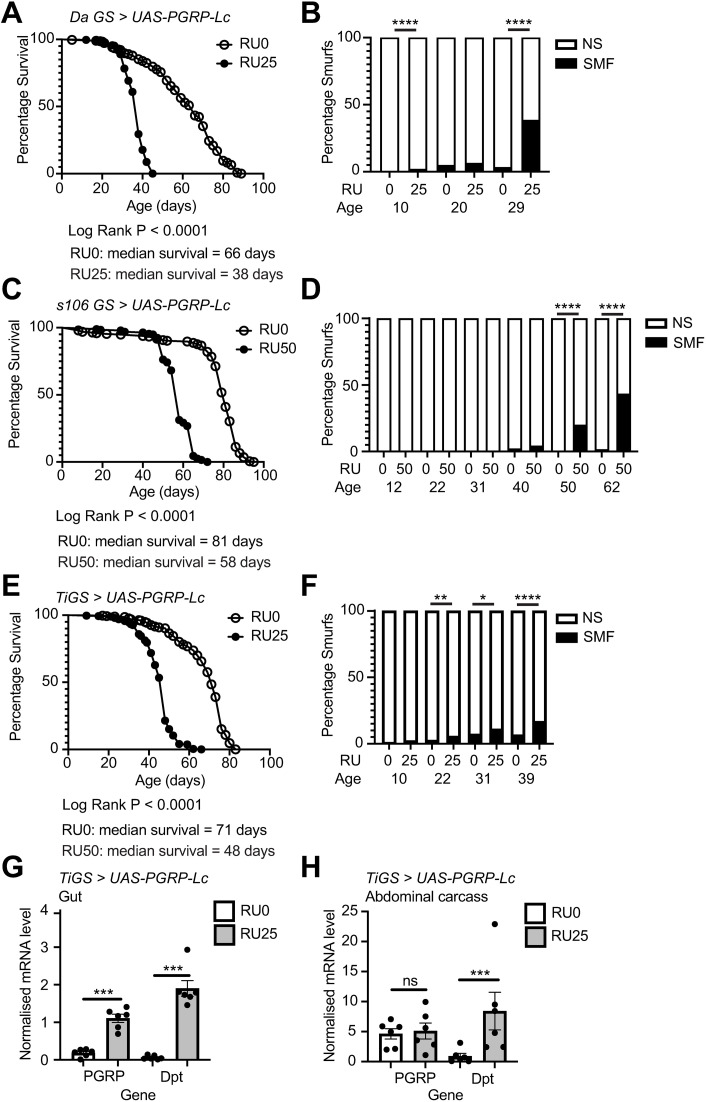
Intestinal *PGRP-Lc* overexpression drives early mortality and intestinal barrier loss. (A-F) Lifespan curves (A, C and E) and Smurf proportions (B, D and F) for *DaGS > UAS-PGRP-Lc* (A and B), *S106 GS > UAS-PGRP-Lc* ((C and D) and TiGS > UAS-PGRP-Lc (E and F) female flies drug induced (RU25, RU50) from early adulthood and uninduced controls (RU0). n > 200 flies/condition, NS = non-Smurf, SMF = Smurf. Log rank test was used for survival data and binomial test for Smurf proportions. (G and H) Normalised mRNA level for *PGRP-Lc* and *Diptericin (Dpt)* in dissected whole gut (G) and abdominal carcass samples (H). n = 6 samples, 5 guts/sample and 5 abdomens (gut removed)/sample. Bar graphs show mean ± SEM. Two-way Anova with Tukey’s multiple comparisons. *p < 0.05, **p < 0.01, ***p < 0.001, ****p < 0.0001.

We previously demonstrated that overexpression of *PGRP-Lc* with the drug-inducible geneswitch driver 5966 drives mortality and loss of intestinal barrier function ( [14], S1A and S1B Figs). PGRP-Lc is a receptor for the Immune Deficiency (IMD) pathway and its overexpression drives immune activation. 5966 drives *UAS-PGRP-Lc* expression in the intestine and in other tissues (S1D and S1E Figs) in adult females. This results in IMD activation in multiple tissues, measured here with qPCR of the antimicrobial peptide Diptericin (S1F and S1G Figs). To further assess the impact of *PGRP-Lc* overexpression in different tissues we compared the ubiquitous geneswitch driver DaGS, the fat body and gut geneswitch driver s106, and the gut specific geneswitch driver TiGS. We found that *PGRP-Lc* overexpression drove early mortality and loss of intestinal barrier function in each case (Figs 1A-F, S1C Fig). Given that gut specific overexpression showed similar mortality to ubiquitous overexpression we conclude that *PGRP-Lc* overexpression in the intestine is sufficient to drive these phenotypes.

However, while *PGRP-Lc* overexpression was restricted to the gut in *TiGS > UAS-PGRP-Lc* flies (Figs 1G and H), Diptericin expression was nevertheless strongly induced outside the gut (Figs 1G and H). Previous work has shown communication between local and systemic immune responses [20]. This combined with our findings suggests that immune activation in the gut may always induce a systemic immune response. This would mean that it is not possible to induce immune activation only in intestinal tissue. It also means that doing so would not reflect normal physiology. Given this it is important to note that we cannot distinguish the roles of intestinal and systemic immune activation in driving intestinal barrier loss and subsequent mortality. We have used both the 5966 and TiGS drivers to overexpress PGRP-Lc throughout this study and hereafter both are referred to as PGRP-Lc overexpression in the text. Which driver has been used is clearly stated on the figures and in the figure legends. Both drivers result in immune activation both in the intestine and systemically

### Overexpression of PGRP-Lc and intestinal barrier loss have distinct impacts on microbial growth

**Fig 2:**
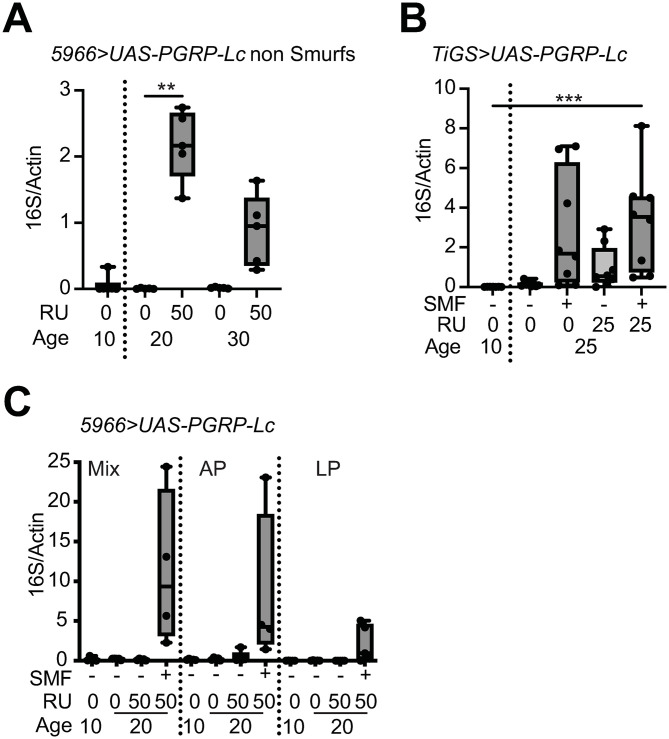
Overexpression of *PGRP-Lc* and intestinal barrier loss have distinct impacts on microbial growth. (A-C) Internal bacterial levels assayed by qPCR of the 16S rRNA gene in surface sterilised females flies drug induced (RU50) from early adulthood and uninduced controls (RU0). n = 6 samples with 5 flies/sample. (A) *5966 > UAS-PGRP-Lc* non-Smurf flies, (B) *TIGS>UAS-PGRP-Lc* non-Smurf and Smurf flies and (C) *5966 > UAS-PGRP-Lc* flies monoassociated with *Acetobacter pasteurianus* (AP), *Lactiplantibacillus plantarum* (LP) or associated with a mix of both species. Boxplots show display the 25-75^th^ percentiles, with the horizontal bar at the median, and whiskers extending from the minimum to maximum points. Two-way Anova with Tukey’s multiple comparisons. **p < 0.01, ***p < 0.001.

Previous work has shown that Drosophila carry an increased internal microbial load with age and following intestinal barrier loss [4,14,15]. Therefore, we assessed the impact of intestinally induced immune activation, and subsequent systemic immune activation, on microbial load. We found that overexpression of *PGRP-Lc* resulted in elevated internal microbial loads in adult female flies carrying their native microbiota (Figs 2A and B). Importantly, increased microbial load was observed in flies overexpressing *PGRP-Lc* that did not have measurable intestinal barrier loss.

To clarify the impact of *PGRP-Lc* overexpression on different microbial groups we used flies monoassociated with two bacterial species commonly found in the *Drosophila* intestine, *Acetobacer pasteurianus* and *Lactiplantibacillus plantarum*, or associated with a mix of both species. Interestingly, we found that *PGRP-Lc* overexpression did not drive overgrowth of either of these bacterial species in flies that did not have measurable intestinal barrier loss (Fig 2C). As expected from previously published work [4,14,15] flies with confirmed intestinal barrier loss showed elevated internal bacterial loads regardless of which bacteria were present and *PGRP-Lc* expression status (Figs 2B and C). We note that the Smurf assay may not detect low levels of intestinal permeability. This means that the microbial overgrowth recorded prior to measurable intestinal barrier loss could either be driven by immune activation or by the early stages of intestinal barrier loss. Therefore, our data is consistent with two scenarios. Either the impact of *PGRP-Lc* overexpression, i.e., IMD activation, on microbial growth is not the same for every microbial species or strain, or the microbial growth we record is being driven by intestinal barrier loss, the different stages of which have differential impacts on microbial growth. For the latter scenario to explain our data there must be microbial species that overgrow in response to the early stages of barrier loss and others that require barrier loss to progress beyond the measurable threshold before they are able to expand. Given that we have identified two bacterial species that do not overgrow in reponse to IMD activation but do overgrow following intestinal barrier loss, we conclude that measurable intestinal barrier loss results in an environmental change that enables these species to overgrow and is not provided by immune activation.

### Overexpression of PGRP-Lc drives intestinal barrier loss and mortality independent of the microbiota

**Fig 3:**
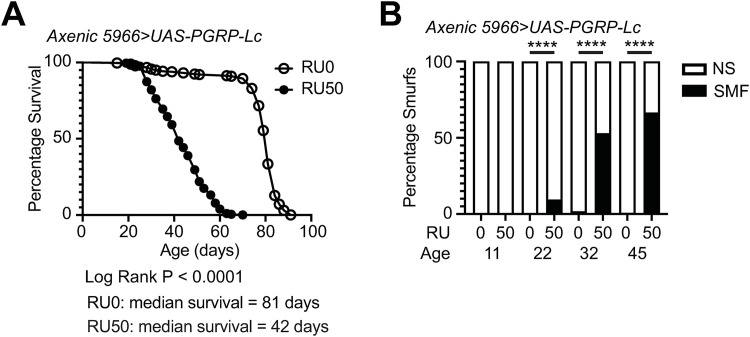
*PGRP-Lc* overexpression drives early mortality and intestinal barrier loss independent of the microbiota. (A and B) Lifespan curves (A) and Smurf proportions (B) for axenic *5966 > UAS-PGRP-Lc* female flies drug induced (RU50) from early adulthood and uninduced controls (RU0). n > 200 flies/condition, NS = non-Smurf, SMF = Smurf. Log rank test was used for survival data and binomial test for Smurf proportions. *p < 0.05, **p < 0.01, ***p < 0.001, ****p < 0.0001.

To test the impact of microbial overgrowth on the reduced lifespan and early intestinal barrier loss that results from *PGRP-Lc* overexpression and subsequent immune activation first in the intestine and then systemically, we assessed these phenotypes in axenic, or germ-free, flies. Axenic flies overexpressing PGRP-Lc showed increased mortality and loss of intestinal barrier function like that of their conventionally reared counterparts (Figs 3A and B). While axenic flies overexpressing PGRP-Lc showed a slightly increased median survival (42 days, Fig 3A) compared to the conventionally reared cohort (36 days, S1A Fig) axenic flies showed a greater difference in median survival between control and treated flies (39 days) than conventionally reared flies (30 days). There were also similar proportions of flies that had lost intestinal barrier function in axenic and conventional flies overexpressing PGRP-Lc at 20 and 30 days of age (~10 and 50% respectively, S1B Fig and Fig 3B).

To ensure that RU486 treatment was not itself driving these phenotypes we assessed mortality and intestinal barrier loss following RU486 treatment in 5966 > w^1118^ flies. We found RU486 treatment had no consistent impact on survival or intestinal barrier loss in conventionally reared or axenic cohorts (S2 Fig). These data demonstrate that *PGRP-Lc* overexpression rather than RU486 treatment drives mortality and intestinal barrier loss. We conclude from these data that the impact of *PGRP-Lc* overexpression on mortality and intestinal barrier loss is independent of the microbiota.

### Overexpression of PGRP-Lc impacts broad readouts of intestinal physiology

**Fig 4:**
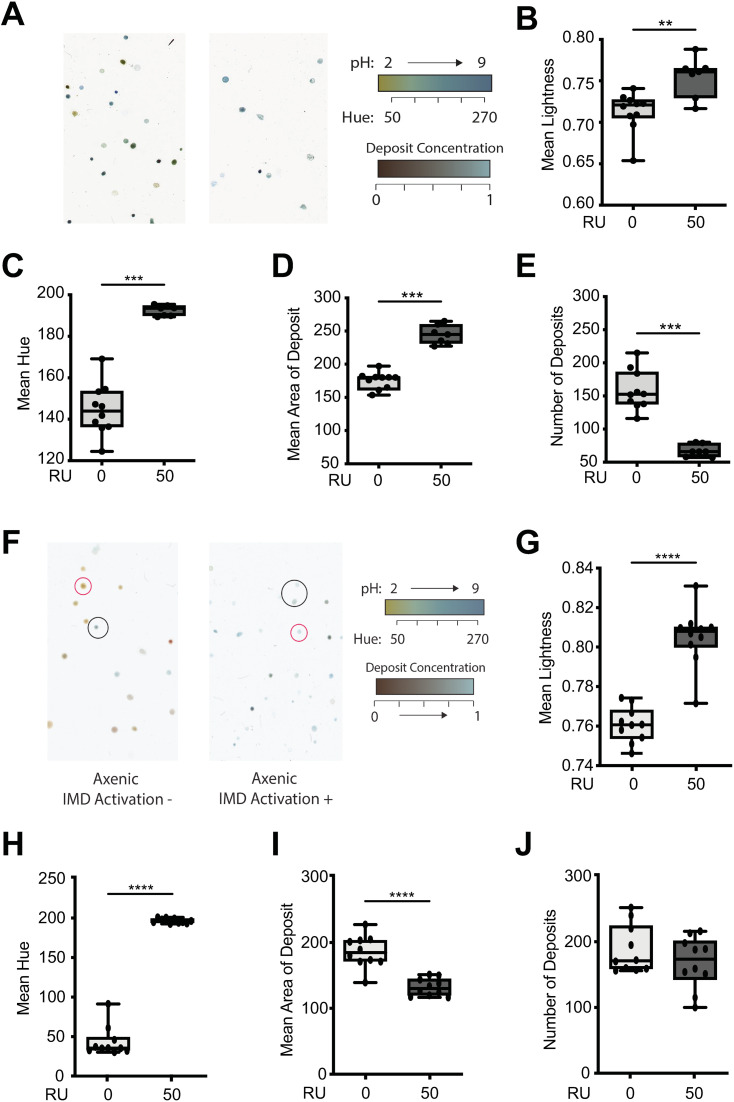
*PGRP-Lc* overexpression impacts intestinal physiology prior to barrier loss. (A – E) Representative T.U.R.D plate images (A) and analysis of faecal output (B-E) from conventionally reared *5966 > UAS-PGRP-Lc* females at 30 days of age drug induced (RU50) from early adulthood and uninduced controls (RU0). (B) Mean lightness, (C) Mean hue, (D) Mean deposit area and (E) Number of deposits. (F – J) Representative T.U.R.D plate images (F) and analysis of fecal output (G-J) from axenic *5966 > UAS-PGRP-Lc* females at 30 days of age drug induced (RU50) from early adulthood and uninduced controls (RU0). (G) Mean lightness, (H) Mean hue, (I) Mean deposit area and (J) Number of deposits. n = 10 plates with 10 flies/plate. Boxplots display the 25–75^th^ percentiles, with the horizontal bar at the median, and whiskers extending from the minimum to maximum points. Mann-Whitney U *p < 0.05, **p < 0.01, ***p < 0.001, ****p < 0.0001.

To assess the impact of PGRP-Lc overexpression and subsequent immune activation in the intestine and systemically on intestinal physiology we used The Ultimate Reader of Dung (T.U.R.D.) [21] to profile the faecal deposits from non-Smurf females overexpressing PGRP-Lc compared to controls. We found *PGRP-Lc* overexpression had a significant impact on four measures of faecal output increasing the water content and alkalinity of deposits while reducing numbers of deposits (Fig 4A–E). This demonstrates that *PGRP-Lc* overexpression significantly impacts multiple elements of intestinal physiology prior to driving early intestinal barrier loss.

To assess whether microbial overgrowth in flies overexpressing PGRP-Lc drives these changes in intestinal physiology we repeated T.U.R.D. analysis on faecal matter from axenic individuals overexpressing PGRP-Lc compared to controls. We found that axenic flies overexpressing *PGRP-Lc* showed increases in faecal water content and alkalinity (Fig 4G and H) like conventional flies (Fig 4B and C). This suggests that the impact of *PGRP-Lc* overexpression on these measures is independent of the microbiota. However, in contrast to conventional flies, axenic flies showed a decrease in faecal deposit area and no significant change in deposit number (Fig 4I and J). This suggests that some aspects of the physiological changes that result from PGRP-Lc overexpression are driven by changes in the microbial population while others are independent.

### PGRP-Lc overexpression suppresses cell junction gene expression and drives stem cell proliferation

**Fig 5:**
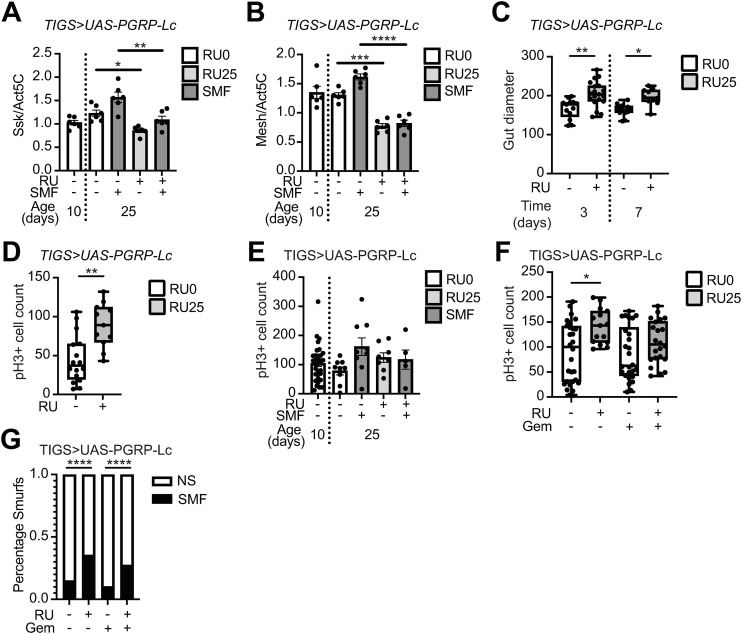
PGRP-Lc overexpression suppresses cell junction gene expression and drives stem cell proliferation. (A & B) Normalised mRNA level for Snakeskin (A, *Ssk)* and Mesh (B) in dissected whole gut samples from *TiGS > UAS-PGRP-Lc* non-Smurf and Smurf females fed RU486 and controls. n = 6 samples, 5 guts/sample. (C & D) Gut diameter at days 3 and 7 (C) and pH3 + cell counts at day 7 (D) post RU486 feeding from *TiGS > UAS-PGRP-Lc* non-Smurf females. (E) pH3 + cell counts at 25 days of age (15 days post RU486 feeding) from *TiGS > UAS-PGRP-Lc* non-Smurf and Smurf females fed RU486 and controls. N = (F & G) pH3 + cell counts in non-Smurf *TiGS > UAS-PGRP-Lc* female midguts at day 7 post RU486 and Gemcitabine feeding (F) and Smurf proportions at 15 days post RU486 and Gemcitabine feeding (23 days old) (G).Bar graphs show mean ± SEM. Boxplots display the 25–75^th^ percentiles, with the horizontal bar at the median, and whiskers extending from the minimum to maximum points. Two-way Anova with Tukey’s multiple comparisons (A, B & E). Mann Whitney U (C & D) Kruskal-Wallis with Dunn’s post hoc test (F) Binomial test (G) *p < 0.05, **p < 0.01, ***p < 0.001, ****p < 0.0001.

Previous work has shown intestinal barrier loss is accompanied by disruption in cell junction gene expression. Genetic knockdown of cell junction gene expression is sufficient to drive intestinal barrier loss, and the epithelial dysplasia associated with it [15,22–24]. To assess the impact of intestinally induced immune activation and subsequent systemic immune activation on these phenotypes we first measured expression levels of the cell junction genes Snakeskin (SSK) and Mesh in *TiGS > UAS-PGRP-Lc* flies both before and after the onset of intestinal barrier loss. We found that *PGRP-Lc* overexpression reduced the expression levels of both SSK and Mesh in the intestines of 25-day old flies (Fig 5A and B). Importantly, we found suppression of SSK and Mesh expression in the intestines of flies that had not yet lost intestinal barrier function as well as those that had (Fig 5A and B). This demonstrates reduced expression of Snakeskin and Mesh occurs prior to the loss of intestinal barrier function in flies overexpressing *PGRP-Lc*.

Induction of intestinal barrier loss through knockdown of Snakeskin has been shown to cause increased gut diameter and to drive stem cell proliferation [15]. Therefore, we assessed the impact of *PGRP-Lc* overexpression on these phenotypes. We found an increased gut diameter in flies overexpressing *PGRP-Lc* compared to controls following just three days of treatment with RU486 (Fig 5C). Further, we found that *PGRP-Lc* overexpression resulted in increased cell proliferation, measured by increased phospho-Histone H3 positive (pH3+) cell numbers, in the intestine following 7 days of RU486 treatment (Fig 5D). No significant increase in cell proliferation was seen at later timepoints (Fig 5E), suggestive of stem cell exhaustion. Stem cell exhaustion has been reported in aged flies [25].

Given the close association between stem cell proliferation, the development of epithelial dysplasia, and the onset of intestinal barrier loss we wanted to assess whether stem cell over proliferation was required for intestinal barrier loss. To do this we fed *PGRP-Lc* overexpressing flies the chemotherapeutic drug Gemcitabine, which has previously been shown to limit cell proliferation in the Drosophila gut in a tumorigenesis model [26]. Following 7 days of *PGRP-Lc* overexpression accompanied by Gemcitabine feeding we found a significant increase in cell proliferation only in *PGRP-Lc* overexpressing flies that were not fed Gemcitabine (Fig 5F); however, we note the variability in our data. We found no significant impact of gemcitabine feeding on the onset of intestinal barrier loss (Fig 5G). Given the minimal suppression of cell proliferation by gemcitabine feeding evident from our data we are unable to conclude that further suppression of cell proliferation would not impact the onset of intestinal barrier loss.

### Both JNK and NFkB signalling can drive intestinal barrier loss and mortality

**Fig 6:**
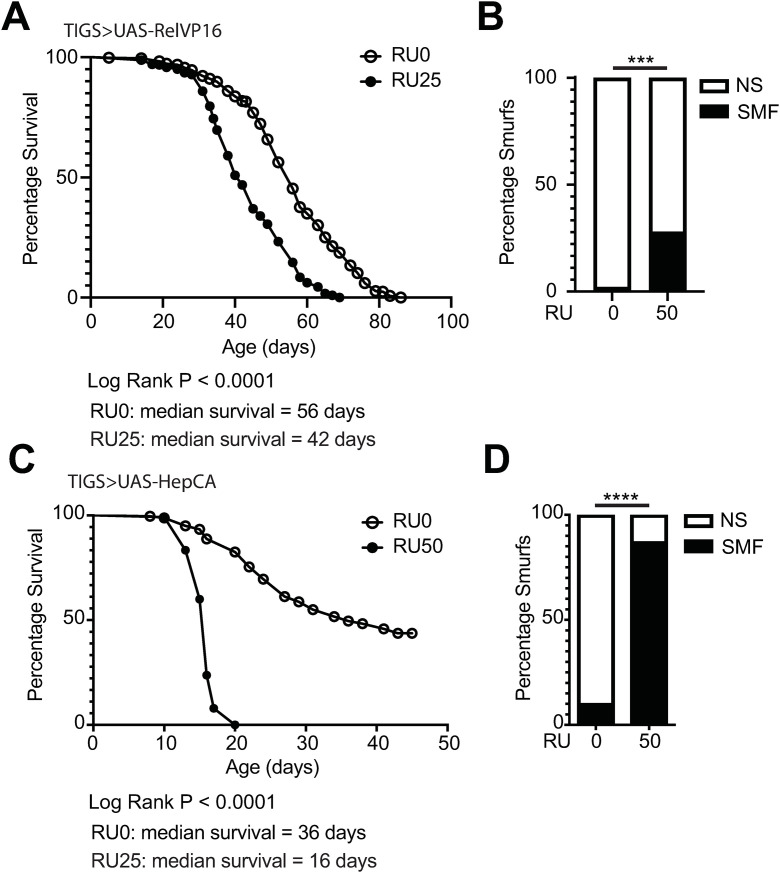
Both Relish and JNK activation drive early mortality and intestinal barrier loss. **(A – D)** Lifespan curves (A & C) and Smurf proportions (B & D) for *TiGS > UAS-RelVP16* (A & B) and *TiGS > UAS-hep*^*CA*^ (C & D) female flies drug induced (RU25 or 50) from early adulthood and uninduced controls (RU0). n > 200 flies/condition, NS = non-Smurf, SMF = Smurf. Log rank test was used for survival data and binomial test for Smurf proportions. *p < 0.05, **p < 0.01, ***p < 0.001, ****p < 0.0001.

Like mammalian Tumor Necrosis Factor (TNF), IMD signalling activates both its downstream NFκB transcription factor Relish and the adjacent Jun N-terminal Kinase (JNK) signalling pathway. The JNK pathway branches from the IMD pathway and contributes to cell proliferation and apoptosis in response to tissue damage caused by stress [27] Crosstalk between the two pathways occurs via a shared intermediary kinase [28], allowing for coordinated regulation of these outcomes.This means that the consequences of *PGRP-Lc* overexpression could be due to Relish activity, JNK signalling or both. To assess the impact of Relish and JNK activation on intestinal barrier loss and mortality we expressed constitutively active versions of *Relish* (*RelVP16*) and the Jun kinase kinase *hemipterous* (*hep*^*CA*^) using the TiGS driver. We confirmed activation by measuring expression levels of the target genes *Diptericin* (Relish) and *Kay* (JNK) (S3 Fig). We found that both *TiGS > UAS-RelVP16* and *TiGS > UAS-hep*^*CA*^ flies showed early mortality (Figs 6A and C) and intestinal barrier loss (Figs 6B and C). The impact of *UAS-hep*^*CA*^ expression was particularly pronounced and led to rapid mortality and intestinal barrier loss like that reported for *Ssk* knockdown [15].

## Discussion

In this study we have clarified the relationship between innate immune signalling, intestinal barrier failure, microbial dysbiosis, epithelial dysplasia and mortality. Strikingly, by distinguishing the effects of immune activation before and after barrier failure we have shown that chronic immune activation is surprisingly well tolerated. The onset of measurable intestinal barrier loss is associated with changes to the microbiota that do not occur with immune activation alone, demonstrating the distinct impact of barrier loss on the microbial population.

### Intestinal immune activation is physiologically inseperable from systemic immune responses

In this study we have demonstrated that genetic induction of immune signalling restricted to intestinal tissue nevertheless drives immune activation outside of the gut. Wu et al (2012) demonstrated that intestinal infection of *Drosophila* initiated an antimicrobial response in the fat body, a tissue analogous to the mammalian liver [20]. In agreement with earlier work demonstrating that feeding a nitric oxide (NO) donor induced antimicrobial peptide (AMP) expression in the fat body [29], this interorgan communication was dependent on intestinal production of reactive oxygen species (ROS) and NO [20]. This response required Relish [20]. Further, these studies support a role for hemocytes, circulating immune cells analogous to macrophages, in the relay of intestinal ROS/NO signals to the fat body [20,29]. Studies in the mosquito have also demonstrated that local and systemic immune responses are tightly linked and support a role for NO and hemocytes in interorgan immune regulation [30]. In mammalian immune responses, interorgan communication has been extensively studied where unresolved local inflammation drives the systemic acute phase response [31]. While they may not have the same molecular basis across different organisms, the physiological links between local and systemic immune responses are evolutionarily conserved and are critical to immune defence.

We set out to determine the impact of local, intestinal immune activation; however, the physiological integration of local and systemic immune signalling has prevented us from doing so. Even if it were technically possible to sever the physiological links between local and systemic immune responses to study them in isolation, doing so would not reflect normal physiology. We emphasise that our intervention is gut specific and the systemic immune activation we measure is driven downstream of the local immune activation that we induce. The outcome is then dependent on local immune activation that may act directly or indirectly, through induction of systemic immune activation, on downstream phenotypes.This means that we must consider the potential impact of systemic immune activation on the intestine in the interpretation of our data.

It is clear from several previous studies that systemic immune activation impacts intestinal regeneration through regulation of intestinal stem cell proliferation. Induction of intestinal regeneration has been shown to be important in the systemic response to wounding [32]. Preventing the intestinal response to a distant wound site worsens survival [32]. Release of the cytokine Unpaired 3 (Upd3) from hemocytes following wounding of the *Drosophila* cuticle induces intestinal stem cell proliferation and intestinal AMP expression [33].

Interestingly, hemocytes are required for the normal regenerative response of the intestine to damage following local oxidative stress or infection [34]. This demonstrates that integration of signals from distinct tissues is important in the regulation of apparently local processes. This strengthens the argument that we cannot and should not consider tissues in isolation. Liu and Jin (2017) review organ-to-organ communication in *Drosophila* more broadly [35]. The involvement of interorgan communication in the onset of intestinal barrier loss, regulation of the intestinal microbiota and in age-related decline more broadly has not yet been studied.

### Intestinal barrier failure and immune activation have distinct impacts on the intestinal environment and how it supports microbial growth

Previous studies in flies and mice have demonstrated that immune suppression can prevent age-related microbial dysbiosis [6,17,18]. Conversely, immune activation has been shown to drive microbial dysbiosis [16,19]. However, the contribution of intestinal permeability to microbial dysbiosis in this context has not been established. Interestingly, a previous study in *Drosophila* demonstrated that acute intestinal IMD activation for 7 days did not result in significant changes to the microbial population [36]. This suggests that the alterations to the intestinal microbiota that are correlated with age-related immune activation may not be directly driven by immune activation but may rather result from decline of the intestinal epithelium.

Here we have addressed this by comparing microbial loads following IMD activation both before and after measurable intestinal barrier failure. We find that flies carrying their native microbiota do show increased microbial loads following ten days of immune activation and prior to measurable intestinal barrier loss. However, microbial loads show a further increase in flies with confirmed barrier failure. Interestingly, in axenic flies reassociated with Lactiplantibacillus plantarum and/or Acetobacter pasteurianus the microbial population does not respond to IMD activation and only shows overgrowth following confirmed intestinal barrier failure. Taken together with previous studies our data suggest that the impact of IMD activation on the microbial population is not acute and that different species/strains may respond to it differently. Further, we note that small increases in intestinal permeability may not be detected by the Smurf assay. This means that individuals that we have identified as non-Smurf may have partial intestinal barrier failure below the detectable threshold. Indeed, expression of barrier components show detectable changes prior to measurable barrier loss. Therefore, we cannot be certain that the microbial overgrowth we record in these individuals is solely due to immune activation, it may also be driven by the early stages of barrier loss.

While the contribution of immune activation to microbial overgrowth in our flies remains unclear, our data further demonstrate the impact of intestinal barrier loss on the microbiota. We have identified two bacterial species that do not respond to IMD activation, or early barrier loss below the detectable threshold, but do overgrow following confirmed intestinal barrier loss. This indicates that barrier loss above the detectable threshold has a distinct impact on the environment in the intestinal lumen and provides conditions for microbial growth that are not provided by immune activation or the early stages of barrier loss. This aligns with previous work demonstrating that while the microbial population shows some age-related changes prior to measurable intestinal barrier loss, the largest changes, particularly to microbial load, occur after confirmed intestinal barrier failure [14]. In our model barrier failure doesn’t result in a worsening of immune activation but does drive further change to the microbial population. This supports the suggestion that much of the impact of immune activation on the microbial population may be through its impact on the intestinal barrier.

We have not established to what extent species in the native microbiota of our model differ in their responses to immune activation and intestinal barrier loss. Confirming the relative contributions of immune activation and intestinal barrier permeability to the onset of microbial dysbiosis will require studies that have greater sensitivity to levels of intestinal permeability than that afforded by the Smurf assay. It will be important for future work to establish to what extent different microbes respond differently to immune activation and intestinal barrier loss. The identification of microbes that overgrow in response to immune activation or the earliest stages of barrier loss may identify targets for early intervention aimed at preventing the development of dysbiosis and associated chronic inflammatory conditions. An understanding of the distinct impacts that immune activation, the onset of barrier loss, and barrier failure have on the environment in the intestinal lumen and how these contribute to the growth of different microbes will be essential to build a full understanding of the drivers of microbial dysbiosis in the inflammed and aged intestine.

### Innate immune signalling drives barrier failure and death in the absence of the microbiota

Several studies have demonstrated that reducing immune activation [17,18] or removing the microbiota [6,14] improves both intestinal barrier function and survival. However, these studies have not addressed the relative impacts of immune activation or microbial dysbiosis on the intestinal barrier because manipulating one also impacts the other. Here we demonstrate that chronic immune activation results in intestinal barrier failure and death even in the absence of the microbiota. The role of immune activation in driving tissue damage and mortality is supported by recent work in a *Drosophila* tumour model that demonstrated that IMD activation resulting from bacterial translocation from the intestines of tumour hosts drove shortened lifespan [37]. Genetic knockdown of the IMD receptor PGRP-Lc specifically in nephrocyte cells was sufficient to extend lifespan [37]. This suggests that targeting immune activation directly, without removing the microbiota, may be sufficient to improve health outcomes following intestinal barrier loss.

We do not know whether acute immune activation results in small increases in intestinal permeability that cannot be detected by the Smurf assay. However, it’s important to note that measurable intestinal permeability does not arise immediately after immune activation. Instead, the onset of measurable intestinal permeability retains its close association with the onset of mortality in our *Drosophila* model. This association between the Smurf phenotype and mortality has been well documented in *Drosophila* and other model systems [4,5,14,15]. This delay in the onset of measurable intestinal permeability suggests that this phenotype may result from ongoing epithelial damage.

### Immune activation drives intestinal stem cell proliferation prior to intestinal barrier failure

The mechanisms by which immune activation drives intestinal permeability remain unclear. One hypothesis is that this occurs through the impact of immune activation on intestinal stem cell (ISC) proliferation and subsequent epithelial dysplasia. Recent studies have demonstrated that loss of cell-cell contacts is sufficient to drive stem cell proliferation [15,22–24] and disrupt cell differentiation [23,24,38]. In addition, it has been shown that septate junction components regulate Yorkie signalling and their loss enables Yorkie to drive expression of Unpaired 3 (Upd3) to promote ISC proliferation [39]. IMD activation can also drive ISC proliferation and disrupt cell differentiation [36,40,41] and supressing IMD activation can reduce ISC proliferation [18,42,43].

Here, we further demonstrate that IMD activation induces ISC proliferation at an early timepoint, significantly preceding the onset of intestinal barrier loss. It is possible that this hyperproliferation leads to an accumulation of misdifferentiated cells, as is seen in the aged intestine [44]. Cell junctions are dynamic as new daughter cells differentiate and integrate into the epithelium [45]. Therefore, accumulation of misdifferentiated cells may impact cell junction formation and barrier function. Whether cell proliferation and subsequent dysplasia are necessary for intestinal barrier failure remains an important question.

In addition to over proliferation of ISCs and disrupted cell differentiation, aberrant enterocyte shedding may also be a potential driver of epithelial damage that could cause barrier loss. It has been shown that infection induced enterocyte shedding requires IMD and activation of IMD signalling is sufficient to induce shedding [46]. Enterocyte shedding was also accompanied by increased ISC proliferation [46], however, the impact of this rapid epithelial turnover on cell-cell contacts and barrier function is unknown.

### Both NFκB and JNK signalling can drive intestinal barrier failure and mortality

It has been well established that JNK activity drives age-related ISC proliferation in *Drosophila* [44,47] and is important for microbial induction of proliferation [48]. Inhibition of JNK has been shown to reduce ISC proliferation following genetic induction of intestinal barrier failure [24] or dextran sodium sulfate (DSS) induced inflammation [49]. Following DSS treatment and in a tumorigenesis model inhibition of JNK also improved intestinal barrier function [49,50]. JNK has also been shown to regulate intestinal barrier function in *C. elegans* [51]. Further, both JNK and IMD signalling can induce enterocyte cell shedding and inhibition of JNK reduces cell shedding following infection [46]. That JNK signalling regulates both ISC turnover and enterocyte shedding likely explains the rapid onset of intestinal barrier failure and death when JNK is constitutively activated.

The role of JNK activity following activation of IMD signalling is complicated by the fact that Relish is a negative regulator of JNK [52,53]. This may explain the difference in response to constitutively active IMD versus JNK, whereby IMD activation appears well tolerated for some time before the onset of intestinal barrier failure and mortality. There are studies that have distinguished the roles of JNK and Relish activity following constitutive activation of IMD. For example, remodelling of *Drosophila* airways following IMD activation was dependent on JNK not Relish [54]. Further, ISC proliferation induced by *PGRP-Lc* overexpression has previously been shown to be dependent on Relish [46].

There is also evidence that JNK and Relish (NFκB) may work cooperatively in some contexts, e.g., enterocyte cell shedding [46]. How both JNK and Relish contribute to age-related intestinal permeability warrants further investigation.

## Conclusions

Our work demonstrates that animals are surprisingly resilient to chronic immune activation, in a way that they are not to the acute induction of intestinal barrier failure. Immune-induced intestinal barrier failure is more closely associated with the onset of mortality than with the onset of immune activation. Further, we add to building evidence that intestinal permeability is a key driver of microbial dysbiosis through mechanisms that are independent of and distinct from its impact on inflammation. Preventing excessive inflammation is critical for health maintenance and has the potential to prevent or delay age onset intestinal permeability, as well as to address the consequences of a leaky gut. However, the impact of intestinal permeability on health needs to be considered independently of its relationship with inflammation.

## Materials and methods

### Fly stocks

This work used the TiGS-2-GeneSwitch (GS) [55], 5966-GS [56], Daughterless-GS (DaGS), S106-GS (BDSC #8151) driver lines, UAS-RelVP16 (Bloomington Drosophila Stock Center (BDSC) #36547), UAS-Hep^CA^ (BDSC #9306, gift of Marc Dionne), UAS-PGRP-Lc (BDSC #30919) and w^1118^.

### Fly culture

Flies were maintained in either vials or bottles in incubators with 65% humidity and a 12-hour light/dark cycle, at either 18°C or 25ºC. Stocks were maintained on cornmeal medium comprised of 1% agar (SLS, FLY1020), 3% dried inactivated yeast (Genesee Scientific, 62–106), 1.9% sucrose (Duchefa Biochemie, S0809), 3.8% dextrose (Melford, G32040) and 9.1% cornmeal (SLS, FLY1110), all given in wt/vol, and water at 90% of the final volume. Preservatives; 10% Nipagin (SLS, FLY1136) in ethanol and an acid mixture comprised of 4.15% phosphoric acid (VWR, 20624.262) and 41.8% propionic acid (Sigma, P5561) in water were added following boiling.

### Lifespan

All experiments were carried out using age-matched, mated females, housed at a density of 27–32 flies per vial. These flies were flipped onto new food within 24 hours of eclosing. They were then allowed a further 24–48 hours to mate before the mated females were sorted into vials under light CO_2_ anaesthesia. Flies were flipped to fresh food every 2–3 days and the number of dead flies was counted. At least 8 vials of 30 females were used per lifespan. To control for bacterial growth on the food, control and experimental flies are always transferred to fresh food at the same time points.

Mifepristone or RU-486 (Cayman chemicals, 10006317), hereafter referred to as RU, food was prepared by the addition of 125 µL of 20 mg/ml RU stock solution per 100mL food for a final concentration of 25 µg/ml (RU25). To ensure sufficient volume for effective mixing 125 µL of RU stock was added to 125 µL of 100% ethanol per 100 mL of food prior to addition to the food. For a concentration of 50 µg/ml (RU50) 250 µL of RU stock solution was added per 100 ml food. Control food was prepared by the addition of 250 µL ethanol per 100 mL food. Food was mixed thoroughly to ensure a homogenous solution.

Gemcitabine (Merck, APO456787943) was prepared at a concentration of 100 µM in water. 50 µl of this solution was added to the surface of standard cornmeal food and allowed to dry for 24 hours in the dark before use.

### Smurf assay

The smurf assay [4] for intestinal barrier integrity was performed by flipping flies onto food containing 2.5% FD&C blue food dye #1 for 24 hours (FastColours, 08BLU00201). After 24 hours, the flies were flipped back onto dye-free food and the number of ‘smurf’ and ‘non-smurf’ flies were recorded.

### Generation of axenic and reassociated flies

To generate sterile (axenic) flies, eggs were collected on apple juice agar plates seeded with a paste of live baker’s yeast in ddH_2_O, then washed and treated with bleach and ethanol as described previously [14]. All steps were carried out in a laminar flow hood. Briefly, < 14 hour-old embryos were dechorionated in 2% sodium hypochlorite solution for 2.5 minutes, rinsed in 70% ethanol for 5 minutes and then rinsed with ~200 mL sterile PBS. Embryos were then resuspended in sterile 0.01% PBSTx (PBS + 0.01% Triton X-100 (Sigma, X100)) and transferred to sterile autoclaved cornmeal medium.

For re-association of sterilised embryos with specific bacteria, we obtained working bacterial cultures by inoculating Lactiplantibacillus plantarum in MRS media and Acetobacter pastiurianus in Mannitol media and culturing at 30ºC for 48 hours. We first established the relationship between optical density at OD600 and bacterial colony forming units (CFU) per volume, to allow us to standardise CFU/fly vial. Bacterial cultures were centrifuged at 2000rpm for 5 minutes, supernatant was removed, and the bacterial pellet was resuspended in sterile 1xPBS. The optical density was adjusted to an OD600 of 0.2. The bacterial solution was then serially diluted 1:5 and plated on the respective bacterial agar. Following incubation for 24–48 hours, colonies were counted from a single dilution sample and this dilution factor was used to calculate CFU/mL. 24-hours prior to embryo collection bacterial cultures prepared as described above and adjusted to the appropriate dilution were used to evenly inoculate vials containing 8 ml of sterile cornmeal medium with 3x10^6^ CFU/vial. Vials inoculated with bacteria were then seeded with sterile embryos. To maintain the integrity of the bacterial population introduced, adult reassociated progeny were handled as though axenic

All medium for sterile and reassociated flies was sterilised by autoclaving for 15 minutes at 121 °C, and all equipment used to push flies was first sterilised in Virkon for at least 20 minutes, then rinsed and stored in 80% ethanol. Gas pads were subsequently dried by running CO_2_ through them. To maintain sterility or reassociation, females were separated into lifespan vials next to an open flame and flipped to new sterile food every 2–3 days in a laminar flow hood.

### Bacterial DNA extraction

30 whole flies were sorted under light CO_2_ anaesthesia into microcentrifuge tubes and frozen at −80 °C. Flies were then surface sterilised in 70% ethanol for 5 minutes and rinsed twice in sterile PBS. 5 flies were sorted into sterile microcentrifuge tubes and homogenised in 200 µl lysis buffer (1 X TE, 1% Triton X-100, 1/100 proteinase K (NEB, P8107S)) using a mechanical pestle, with a further 300 µl of lysis buffer being added after homogenisation. Lysate was incubated for 1.5 hours at 55 °C then transferred to screw cap vials containing 0.4 g sterilised low binding 100-micron silica beads (OPS Diagnostics, BLBG 100-200-11) and vortexed for 10 minutes at max speed. Lysate was then transferred back to the original tube and incubated for a further 1.5 hours at 55 °C followed by 10 minutes at 95 °C.

### RNA extraction

Total RNA was extracted from 5 midguts per biological replicate. Midguts were dissected in ice-cold PBS and included the cardia and midgut, through to the midgut-hindgut junction, excluding the hindgut and Malpighian tubules. Midguts were homogenised in 100 µL TRIzol™ (Thermofisher, 15596026) using a motorised pestle. RNA was extracted following the standard TRIzol protocol.

### cDNA preparation

For a 10 µL reaction, 1 µL DNase buffer (Thermo, EN0521) and 0.5 µL DNase 1 (Thermo, EN0521) were added to 8.5 µL RNA solution and incubated for 30 minutes at 37 °C, to remove any DNA contaminants. The DNase enzyme was then deactivated by the addition of 0.5 µL 50mM EDTA (Thermo, EN0521) and subsequent incubation for 10 minutes at 65 °C. cDNA synthesis was carried out using Thermo Scientific’s First Strand cDNA Synthesis kit. In short, 1 µL of random hexamers (Thermo, SO142) were added and the samples were incubated at 70 °C for 5 minutes before a 9 µL of a master mix containing 4 µL first strand buffer (Thermo, EPO442), 0.5 µL RIbolock RNase inhibitor (Thermo, EO0382), 2 µL 10mM dNTPs (Thermo, R0181), 0.2 µL RevertAid reverse transcriptase (Thermo, EPO442) and 2.3 µL molecular grade water (Sigma, W4502) per well, was added. The samples were then incubated for 10 minutes at room temperature, followed by 1 hour at 37 °C and 10 minutes at 70 °C. The samples were spun following each incubation step.

### Quantitative PCR

qPCR was performed with Power SYBR™ Green master mix (Fisher, 10219284) on a Bio-RAD CFX Connect Real-Time PCR detection system. Either 4.5 µL bacterial DNA or 1 µL of *Drosophila* cDNA sample was used in a 10 µL reaction with 5 µL of Power SYBR™ Green and 0.5 µL of L + R primer mix. Any remaining volume was made up with molecular grade water.

Cycling conditions were: 95°C for 10 minutes, then 40 cycles of 95°C for 15 seconds followed by 60°C for 60 seconds. All calculated gene expression values were normalised to the value of a housekeeping gene, either Act5C or rp49.

The primer sequences used to assess gene expression are as follows:

SSK_F – CACTGGATGCCACACCATT
SSK_R – TGGTGTCGCACAGCTCTC [15]
Mesh_F – AGCCCGATCAATACTCAGGA
Mesh_R – CCATATACCAGGCCAGAGGA [15]
Dpt_F – ACCGCAGTACCCACTCAATC
Dpt_R – CCCAAGTGCTGTCCATATCC
PGRP_49F – CCTTCCTGCTGGGTATCGTA
PGRP_49R - CCACCAATGTTGTCCAGATTT
Kay_F – CAGCATCAGCGACAGGATTA
Kay_R – TCTGGCCGGTCTCAAAGTT [50]
Act5c_F – TTGTCTGGGCAAGAGGATCAG
Act5c_R – ACCACTCGCACTTGCACTTTC
Rp49_F – ATCGGTTACGGATCGAACAA
Rp49_R - GACAATCTCCTTGCGCTTCT

Universal primers for the 16S ribosomal RNA gene were against variable regions 1 (V1F) and 2 (V2R) [14]. Levels of the bacterial 16S rRNA gene were normalised to the level of the *Drosophila* Actin gene, as previously published [14].

16S_V1F - AGAGTTTGATCCTGGCTCAG
16S_V2R - CTGCTGCCTYCCGTA
Act_F – TTGTCTGGGCAAGAGGATCAG
Act_R – ACCACTCGCACTTGCACTTTC

### Faecal analysis

Faecal analysis was conducted as described previously [14].

### Dissection and immunofluorescent staining of midguts

Whole guts were dissected into ice-cold PBS, with the crop, cardia, ovaries, and Malphigian tubules removed. Fixation of was carried out with 16% PFA for 30 minutes, followed by three immediate washes in PBS. Blocking buffer (PBS with 0.5% Triton X-100, 0.5% BSA and 5% fresh NGS) was added and incubated for 30 minutes.

Samples were immunostained with primary antibody (rabbit a-pH3, Merck, H0412) at 1:2000 overnight on a rocker at 4°C. The following morning, guts were washed three times for 10 minutes each, in PBS. Samples were incubated with the secondary antibody (Goat anti-rabbit Alexa Fluor 555) at 1:500 on a rocker at room temperature for 2 hours. DAPI (1:2000) was added for a 10-minute incubation to stain nuclei. Guts were again washed three times for 10 minutes each, in PBS. Samples were mounted in VectaShield Antifade Mounting Medium (Vector Laboratories, H-1000–10)

### Microscopy

For the quantification of pH3 + cells, images were acquired with a 10x objective and rhodamine laser under the Zeiss Cell Observer microscope using AxioVision 40 software (Carl Zeiss). The total number of pH3 + cells across the entire length of each gut was counted.

To measure variations in midgut diameter, single images were taken under the10x objective and DAPI laser using the Zeiss Cell Apotome microscope and measured using the AxioVision Rel 4.8 software (Carl Zeiss). Diameter was calculated just above the midgut-hindgut junction for each midgut, as in [15].

### Statistics

All Statistical analyses were performed using GraphPad Prism 9, excluding the binomial tests which were performed in R (Rstudio build 372, R version 4. 0. 3). Survival curve analysis was completed using the log-rank test in GraphPad Prism; the median survival was calculated by Graph Pad Prism 8 and is an approximation to the closest sampling time. Normality of the data was tested by Shapiro-Wilk tests to determine the most appropriate statistical test. Differences between two points were analysed by students t-test, or Mann-Whitney tests for normally and non-normally distributed data respectively. Differences in gene expression values between more than two time points, within or between conditions, were analysed by two-way Anova with Tukeys multiple comparison or Kruskal-Wallis with Dunn’s test for normally and non-normally distributed data respectively. P-values less than 0.05 were considered statistically significant. Bar-graph error bars depict ± standard error of the mean (SEM). For all box-plots shown, boxes display the 25–75^th^ percentiles, with the horizontal bar at the median, and whiskers extending from the minimum to maximum points.

## Supporting information

S1 Fig The 5966 geneswitch drives UAS-PGRP-Lc expression in the gut and other tissues which results in early mortality and intestinal barrier loss. (A and B) Lifespan curves (A) and Smurf proportions (B) for *5966 > UAS-PGRP-Lc* female flies drug induced (RU50) from early adulthood and uninduced controls (RU0). n > 200 flies/condition, NS = non-Smurf, SMF = Smurf. (C) Replicate lifespan curve for *TIGS>UAS-PGRP-Lc* female flies drug induced (RU25) from early adulthood and uninduced controls (RU0). n > 200 flies/condition. Log rank test was used for survival data and binomial test for Smurf proportions. (D-G) Normalised mRNA level for *PGRP-Lc* (D and E) and *diptericin (Dpt*, F and G)) in dissected whole gut (D and F) and body carcass samples (E and G) on different RU486 doses. n = 6 samples, 5 guts/sample and 5 carcasses (gut removed)/sample. Bar graphs show mean ± SEM. Two-way Anova with Tukey’s multiple comparisons. *p < 0.05, **p < 0.01, ***p < 0.001, ****p < 0.0001.
(PDF)

S2 fig RU486 feeding doesn’t consistently drive early mortality or intestinal barrier loss. Lifespan curves (A and C) and Smurf proportions (B and D) for conventional (A and B) axenic (C and D) *5966 > w*^*1118*^ female flies drug fed (RU50) from early adulthood and controls (RU0). n > 200 flies/condition, NS = non-Smurf, SMF = Smurf. Log rank test was used for survival data and binomial test for Smurf proportions. *p < 0.05, **p < 0.01, ***p < 0.001, ****p < 0.0001.
(PDF)

S3 fig Constitutively active RelVP16 and HepCA drive target gene expression. (A and B) Normalised mRNA level for *Diptericin (Dpt)* (A) and *Kay* (B) in dissected whole gut from *TiGs > UAS-RelVP16* (A) and *TiGs > UAS-HepCA* (B) females fed RU486 for 72 hours or controls. n = 6 samples, 5 guts/sample. Bar graphs show mean ± SEM. Boxplots display the 25–75^th^ percentiles, with the horizontal bar at the median, and whiskers extending from the minimum to maximum points. Two-way Anova with Tukey’s multiple comparisons. *p < 0.05, **p < 0.01, ***p < 0.001, ****p < 0.0001.
(PDF)

S1 Data All data used in this article. Data are organised into separate tabs by figure and figure panel.
(XLSX)
