# Supplementary material for: Chronic intestinal immune activation reveals separable impacts of inflammation and barrier loss on hallmarks of ageing
Source: PLoS One. 2026 Feb 13;21(2):e0342910. doi: 10.1371/journal.pone.0342910 (PMC12904396; doi:10.1371/journal.pone.0342910)
Supplement: S1 Fig — (A and B) Lifespan curves (A) and Smurf proportions (B) for 5966 > UAS-PGRP-Lc female flies drug induced (RU50) from early adulthood and uninduced controls (RU0). n > 200 flies/condition, NS = non-Smurf, SMF = Smurf. (C) Replicate lifespan curve for TIGS>UAS-PGRP-Lc female flies drug induced (RU25) from early adulthood and uninduced controls (RU0). n > 200 flies/condition. Log rank test was used for survival data and binomial test for Smurf proportions. (D-G) Normalised mRNA level for PGRP-Lc (D and E) and diptericin (Dpt, F and G)) in dissected whole gut (D and F) and body carcass samples (E and G) on different RU486 doses. n = 6 samples, 5 guts/sample and 5 carcasses (gut removed)/sample. Bar graphs show mean ± SEM. Two-way Anova with Tukey’s multiple comparisons. *p < 0.05, **p < 0.01, ***p < 0.001, ****p < 0.0001. (PDF) [file pone.0342910.s001.pdf]

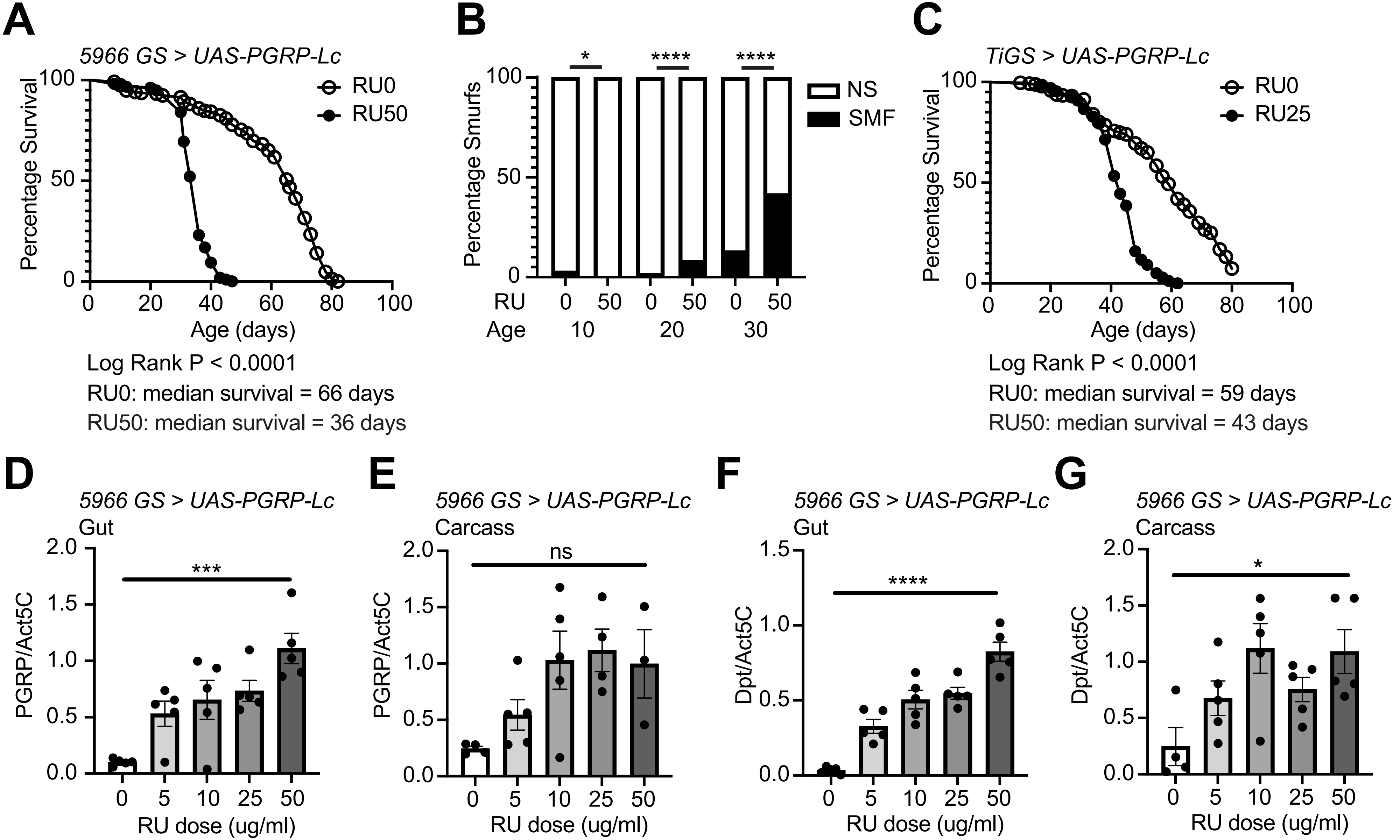
A
5966 GS > UAS-PGRP-Lc
Percentage Survival
RU0
RU50
Age (days)
Log Rank P < 0.0001
RU0: median survival = 66 days
RU50: median survival = 36 days
B
Percentage Smurfs
NS
SMF
RU
Age
C
TiGS > UAS-PGRP-Lc
Percentage Survival
RU0
RU25
Age (days)
Log Rank P < 0.0001
RU0: median survival = 59 days
RU50: median survival = 43 days
D
5966 GS > UAS-PGRP-Lc
Gut
PGRP/Act5C
RU dose (ug/ml)
E
5966 GS > UAS-PGRP-Lc
Carcass
PGRP/Act5C
ns
RU dose (ug/ml)
F
5966 GS > UAS-PGRP-Lc
Gut
Dpt/Act5C
RU dose (ug/ml)
G
5966 GS > UAS-PGRP-Lc
Carcass
Dpt/Act5C
RU dose (ug/ml)
