# Supplementary material for: Chronic intestinal immune activation reveals separable impacts of inflammation and barrier loss on hallmarks of ageing
Source: PLoS One. 2026 Feb 13;21(2):e0342910. doi: 10.1371/journal.pone.0342910 (PMC12904396; doi:10.1371/journal.pone.0342910)
Supplement: S2 fig — Lifespan curves (A and C) and Smurf proportions (B and D) for conventional (A and B) axenic (C and D) 5966 > w1118 female flies drug fed (RU50) from early adulthood and controls (RU0). n > 200 flies/condition, NS = non-Smurf, SMF = Smurf. Log rank test was used for survival data and binomial test for Smurf proportions. *p < 0.05, **p < 0.01, ***p < 0.001, ****p < 0.0001. (PDF) [file pone.0342910.s002.pdf]

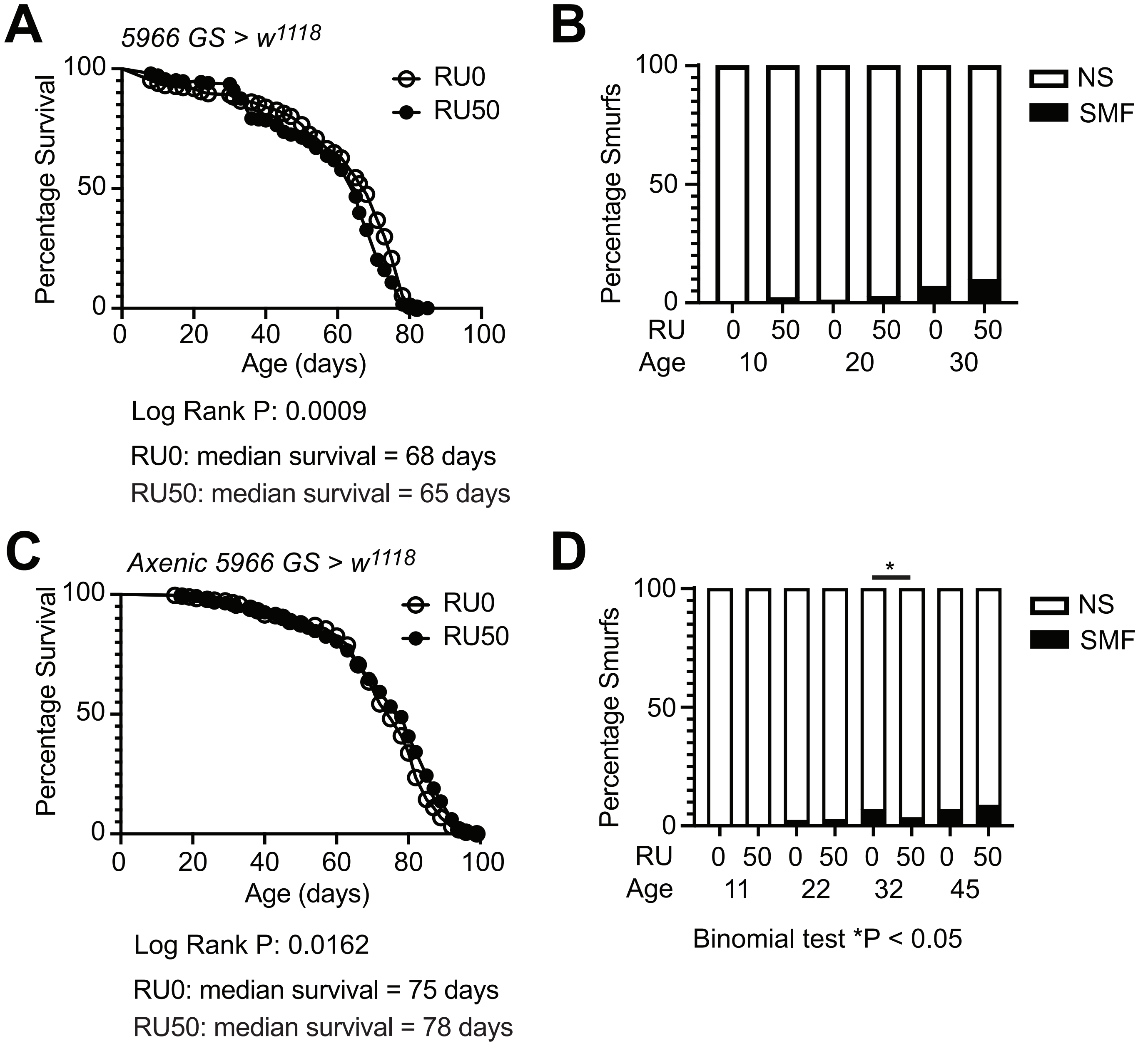
A
5966 GS > w1118
Percentage Survival
100
50
0
0
20
40
60
80
100
Age (days)
RU0
RU50
Log Rank P: 0.0009
RU0: median survival = 68 days
RU50: median survival = 65 days
B
Percentage Smurfs
100
50
0
NS
SMF
RU 0 50 0 50 0 50
Age 10 20 30
C
Axenic 5966 GS > w1118
Percentage Survival
100
50
0
0
20
40
60
80
100
Age (days)
RU0
RU50
Log Rank P: 0.0162
RU0: median survival = 75 days
RU50: median survival = 78 days
D
*
Percentage Smurfs
100
50
0
NS
SMF
RU 0 50 0 50 0 50 0 50
Age 11 22 32 45
Binomial test *P < 0.05
