# Supplementary figures and images for: Chronic intestinal immune activation reveals separable impacts of inflammation and barrier loss on hallmarks of ageing
Source: PLoS One. 2026 Feb 13;21(2):e0342910. doi: 10.1371/journal.pone.0342910 (PMC12904396; doi:10.1371/journal.pone.0342910)

**A**

TIGS&gt;UAS-RelVP16

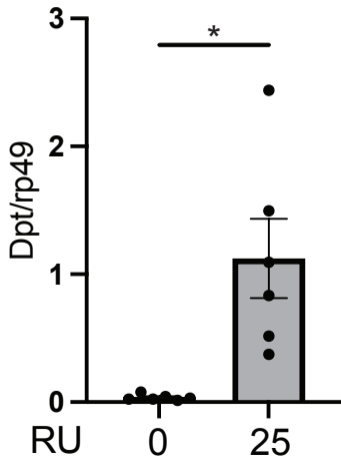**B**

TIGS&gt;UAS-HepCA

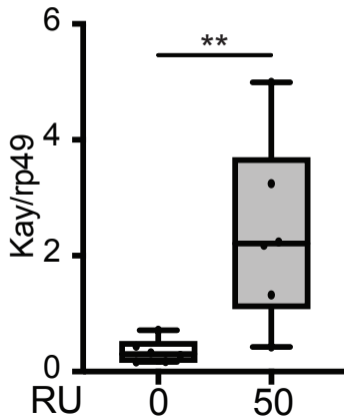

Supplement: S3 fig — (A and B) Normalised mRNA level for Diptericin (Dpt) (A) and Kay (B) in dissected whole gut from TiGs > UAS-RelVP16 (A) and TiGs > UAS-HepCA (B) females fed RU486 for 72 hours or controls. n = 6 samples, 5 guts/sample. Bar graphs show mean ± SEM. Boxplots display the 25–75th percentiles, with the horizontal bar at the median, and whiskers extending from the minimum to maximum points. Two-way Anova with Tukey’s multiple comparisons. *p < 0.05, **p < 0.01, ***p < 0.001, ****p < 0.0001. (PDF) [file pone.0342910.s003.pdf]
